# Perturbation of resting-state network nodes preferentially propagates to structurally rather than functionally connected regions

**DOI:** 10.1038/s41598-021-90663-z

**Published:** 2021-06-14

**Authors:** Davide Momi, Recep A. Ozdemir, Ehsan Tadayon, Pierre Boucher, Alberto Di Domenico, Mirco Fasolo, Mouhsin M. Shafi, Alvaro Pascual-Leone, Emiliano Santarnecchi

**Affiliations:** 1 Berenson-Allen Center for Non-Invasive Brain Stimulation, Beth Israel Deaconess Medical Center, Harvard Medical School, Boston, MA USA; 2 Department of Neuroscience, Imaging and Clinical Sciences, University of Chieti-Pescara, Chieti, Italy; 3 Department of Psychological, Health and Territorial Sciences , University of Chieti-Pescara, Chieti, Italy; 4 Hinda and Arthur Marcus Institute for Aging Research and Deanna and Sidney Wolk Center for Memory Health, Hebrew Senior Life, Boston, MA USA; 5 Department of Neurology, Harvard Medical School, Boston, MA USA; 6 Guttmann Brain Health Institute, Barcelona, Spain; 7 Siena Brain Investigation & Neuromodulation Lab (Si-BIN Lab), Department of Medicine, Surgery and Neuroscience, Neurology and Clinical Neurophysiology Section, University of Siena, Siena, Italy

**Keywords:** Neurology, Brain, Cognitive neuroscience, Neural circuits

## Abstract

Combining Transcranial Magnetic Stimulation (TMS) with electroencephalography (EEG) offers the opportunity to study signal propagation dynamics at high temporal resolution in the human brain. TMS pulse induces a local effect which propagates across cortical networks engaging distant cortical and subcortical sites. However, the degree of propagation supported by the structural compared to functional connectome remains unclear. Clarifying this issue would help tailor TMS interventions to maximize target engagement. The goal of this study was to establish the contribution of functional and structural connectivity in predicting TMSinduced signal propagation after perturbation of two distinct brain networks. For this purpose, 24 healthy individuals underwent two identical TMS-EEG visits where neuronavigated TMS pulses were delivered to nodes of the default mode network (DMN) and the dorsal attention network (DAN). The functional and structural connectivity derived from each individual stimulation spot were characterized via functional magnetic resonance imaging (fMRI) and Diffusion Weighted Imaging (DWI), and signal propagation across these two metrics was compared. Direct comparison between the signal extracted from brain regions either functionally or structurally connected to the stimulation sites, shows a stronger activation over cortical areas connected via white matter pathways, with a minor contribution of functional projections. This pattern was not observed when analyzing spontaneous resting state EEG activity. Overall, results suggest that structural links can predict network-level response to perturbation more accurately than functional connectivity. Additionally, DWI-based estimation of propagation patterns can be used to estimate off-target engagement of other networks and possibly guide target selection to maximize specificity.

## Introduction

Transcranial Magnetic Stimulation (TMS) allows to transiently perturb the brain in vivo in a control and reliable manner ^1^. A compelling way to study the cascade of events induced by an external perturbation is combining TMS with electroencephalography (EEG) which allows to reach a good compromise between high temporal and spatial resolution ^2^. Notably, the effects of a TMS pulse are not constrained to the portion of local grey matter directly engaged by the stimulation pulse, but rather affect distant cortical and subcortical sites as well, connected to the stimulated region by means of long range projections ^3,4^. Indeed, an external magnetic perturbation initially depolarizes the membrane of the superficial neural tissue underneath the coil, causing an action potential, which then spreads along specific anatomical connections ^3,5^. These transsynaptic distant effects have been previously demonstrated to be dependent on the strength and the nature (e.g. inhibitory or excitatory) of the anatomical connections themselves ^6^. Previous animal studies have shown how the TMS pulse preferentially propagates through structural monosynaptic connections, stressing the importance of the underlying anatomical connectivity between regions and the number of synaptic steps in guiding the observed effects ^6^. Nevertheless, the amount of TMS signal propagation cannot be considered to be solely determined by the underlying anatomical connections of the perturbed region, but to also depend on its initial functional state and connectivity profile as well ^7^.

The complexity and organization of the structural and functional connectome can be respectively captured in vivo via diffusion weighted imaging (DWI) and functional magnetic resonance imaging (fMRI) data collected during resting-state (rs-fMRI) ^8^. The acquired functional spontaneous dynamics can be decomposed into separate but integrated “modules” also known as resting state networks (RSNs) ^9,10^. In this context, recent studies have focused on investigating individual RSNs dynamics, demonstrating how the effects of an externally induced network’s node perturbation mainly propagate within distal cortical regions belonging to the same network ^11,12^. In a recent TMS-EEG study ^13^, we have stimulated two RSNs (i.e. Default Mode Network, DMN, and Dorsal Attention Network, DAN) showing how network-level structural connectivity is more relevant than local and global brain properties in shaping TMS signal propagation. However, even though many studies have evaluated the propagation of the TMS-induced action potentials using DWI ^14^ and fMRI ^15^ connectivity metrics, none so far have investigated which of the two techniques is better in capturing the spreading of such TMS-induced signal. Potential differences in the prediction of perturbation patterns via either DWI-based metrics of structural connectivity or rs-fcMRI metrics of functional connectivity, has important fundamental implications regarding the effects of brain stimulation and the significance of the DWI- versus rs-fcMRI-derived connectomes.

In the present study, we initially use fMRI-guided TMS-EEG to selectively perturb one of two neighboring nodes of rs-fcMRI-defined DMN and DAN networks. The DMN and DAN respectively exhibit task-related deactivation and activation during rs-fMRI scanning ^10^. We then characterized the individual structural and functional connectivity profile of the stimulated brain tissue based on biophysical modeling ^16^. For both the DMN and DAN, we evaluated how the TMS pulse propagated to the surrounding nodes based on their structural or functional relationship with the stimulated site. Given that functional connectivity is dependent on physical connection, we hypothesized that DWI-based structural connectivity would be a better predictor of the propagation of TMS effects than the rs-fcMRI-based functional connectivity profile. Additionally, to verify whether propagation patterns observed after stimulation reflect ongoing oscillatory dynamics also present at rest, the same analysis was repeated looking at spontaneous network activity using resting-state EEG recordings which we then compared with the TMS-based perturbation data. Finally, considering the quest for data reproducibility, the same analyses were repeated on data collected on the same sample of healthy individuals across two separate study visits one month apart.

## Material and methods

### Participants

The study was approved by the Institutional Review Board of the Beth Israel Deaconess Medical Center. Each participant gave their written informed consent to the study which conformed to the Declaration of Helsinki and had been approved by the Institutional Review Board of the Beth Israel Deaconess Medical Center. Twenty-four right-handed ^20^ healthy volunteers (mean age = 32 ± 10 years, ranging from 19 to 49 years) with normal neurological and psychiatric evaluation and no history of drugs acting on the central nervous system were recruited through flyers and on-line advertisement. A pre-TMS MRI assessment was carried out comprehensive of a T1-weighted (T1w) anatomical, a resting state fMRI and a DWI scans. Following each participant carried out two TMS visits, separated by one month, where 120 single pulses were delivered in two neighboring parietal nodes corresponding to the DMN and DAN.

The TMS pulses intensity was set at 120% of resting motor threshold (RMT) which was determined at the beginning of each TMS visit according to international TMS guidelines ^21^. Methods for data acquisition are presented in the following paragraph and have been also described in ^12,13^.

### Structural and functional connectivity

**Figure 1:**
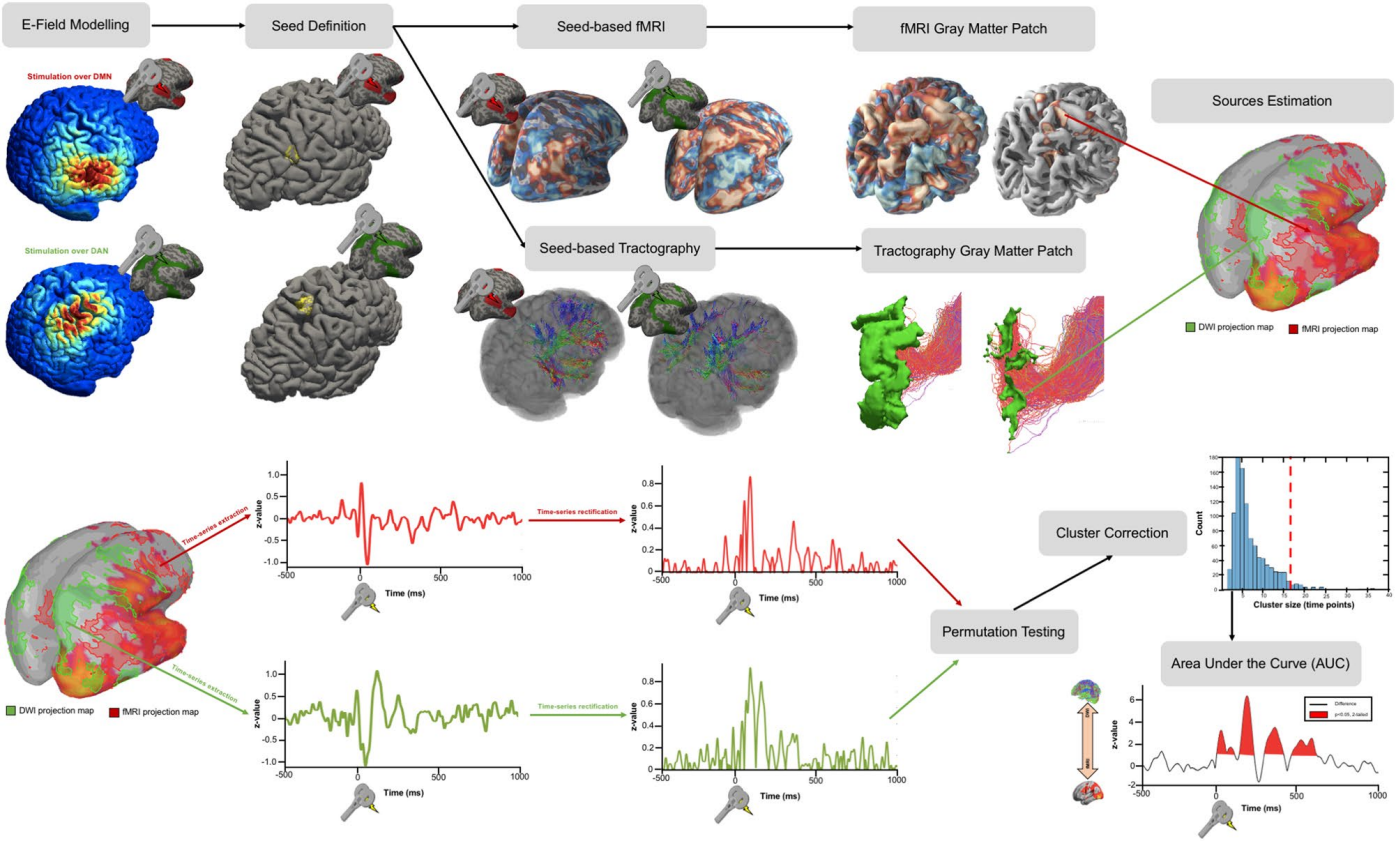
Methodological workflow and features extraction. TMS-induced electric field was modelled with SimNIBS ^16^. Fiber tractography and resting state functional connectivity (rs-FC) were computed from the point with the maximal E-field. As for rs-FC analysis, the final maps were obtained z-transforming, thresholding and FDR-correcting the raw connectivity profile. As for tractography, the reconstructed tract was used to threshold the grey matter map where only the voxels reached by a streamline were retailed. The final rs-FC map and the tract-thresholded gray matter were used to extract the source reconstructed EEG timeseries in order to establish which between functional and structural connectivity mostly predict the TMS-induced signal propagation. The EEG signal was projected at source level using the minimum norm estimation (MNE) method with current density and constraining source dipoles to the cortical surface. The raw time series were first rectified^17^ and then a baseline bootstrapping procedure^18^ was applied. Then, 1000 permutation t-test were performed in which the surrogated DWI vs rs-FC difference was computed after each iteration and statistically compared with the real difference^19^. Finally, the cluster threshold was determined as the 95th percentile of the cluster’s surrogate distribution and the area under the curve (AUC) of the significant clusters was extracted within the 350 ms post-TMS period, with positive and negative values indicating that the TMS-EEG signal propagated more on DWI or fMRI projection map, respectively. For further details on TMS-induced electric field modelling please refer to Supplementary Materials and Methods. For a visualization of the individual white matter bundle of both DAN and DMN please see Supplementary Figs. S2 and S3. No software was used to generate this figure.

As shown in Fig. 1, to identifying the amount of grey matter directly engaged by the external perturbation, the TMS-induced electric field was modelled with SimNIBS ^16^. Given that in literature there is no consensus on how selectively identify only the neural tissue recruited by the TMS pulse ^22^, we defined the point with maximal E-field and from there created a sphere of radius 0.5 cm. Such region of interest (ROI) was used as seed to performed resting state functional connectivity (rs-FC) and fiber tractography in order to characterize the individual functional and structural profile associated with the stimulation spot.

As for rs-FC analysis, the positive correlation coefficients were firstly transformed in Z-scores using Fisher’s transformation and then converted into p-values. In order to address the issue of multiple testing comparisons, a false discovery rate (FDR) correction at p < 0.05 was applied to obtain the final rs-FC map representing the grey matter regions positively correlated with the stimulation spot.

As for structural connectivity, a seed-based anatomically constrained tractography ^23^ was performed from the individual TMS spot to generate a fibers bundle with one million of streamlines of 250 mm maximum length, a backtrack flag and a fiber orientation function cut-off of 0.15. Ventricles and subcortical areas were excluded given that source reconstructed EEG signal cannot be extracted from those regions. The reconstructed tract was used to threshold the grey matter map where only the voxels reached by a streamline were retailed. The remaining voxels represent the cortical regions structurally connected with the stimulation spot.

The final rs-FC map and the tract-thresholded gray matter were used to extract the source reconstructed EEG timeseries in order to establish which between functional and structural connectivity mostly predict the TMS-induced signal propagation. For further details on TMS-induced electric field modelling please refer to Supplementary Materials and Methods. For a visualization of the individual white matter bundle of both DAN and DMN please see Supplementary Figs. S2 and S3.

### MRI data acquisition

The MRI evaluation was performed on a 3 T scanner (GE Healthcare, Ltd., United Kingdom). The T1w was used for neuronavigation and was acquired using a 3D spoiled gradient echo sequence: 166 axial-oriented slices for whole-brain coverage; 240 mm isotropic field-of-view; 0.937-mm × 0.937 mm × 1 mm native resolution; flip angle = 15°; TE/TR ≥ 2.9/6.9 ms; duration ≥ 432 s. rs-fMRI data were acquired in three runs of 5 min each with eyes open, using an axial plane (bottom-up), FOV = 240 mm, TE = 25 ms, TR = 3,196 ms, slice thickness = 2.5 mm, flip angles = 90°, voxel size of 1.87 × 1.87 × 2.5. Finally, DWI sequence were collected using a single-shot echo planar imaging (slices = 71; matrix size = 256 × 256 × 71; voxel size = 0.8 mm × 0.8 mm × 2.2 mm; repetition time = 8500 ms, time echo = 79 ms; 30 non-colinear directions, b-value = 1000 s/mm^2^).

### DWI data preprocessing

A customize pipeline running in Ubuntu 18.04 LTS was used for the preprocessing of DWI images using tools in FMRIB Software Library (FSL 5.0.3; www.fmrib.ox.ac.uk/fsl) ^24^, MRtrix3 (https://www.mrtrix.org/) ^25^, FreeSurfer ^26^ and ANTs (http://stnava.github.io/ANTs/) ^27^. All images were denoised ^28^, preprocessed via FSL's EDDY ^29^, and bias field corrected ^30^. The response function for a single fiber population was estimated using spherical deconvolution Tournier algorithm ^31^. Simultaneously, the T1w images were coregistered to the b0 volume and then segmented using FAST algorithm ^30^.

### TMS

As reported in ^13^, TMS was delivered using a figure-of-eight shaped coil with dynamic fluid cooling (Magspro 75 mm cool B-65, Magpro A/S., Denmark) attached to a MagPro X-100 stimulator (MagVenture A/S, Denmark). T1w anatomical images were imported into the Brainsight™ TMS Frameless Navigation system (Rogue Research Inc., Montreal, Canada), and a coregistration procedure was performed using scalp landmarks (nasion, vertex, and the two preauricular points) in order to monitor the coil position. Motor evoked potentials (MEPs) were recorded with active electrodes positioned on the right first dorsal interosseous (FDI) and the right abductor pollicis brevis (APB) muscles, while the reference electrode was placed over the metacarpophalangeal joint of the index finger. EMG data were amplified and digitized using a Powerlab 4/25 T data acquisition system (ADInstruments) at a sampling rate of 4000 Hz (bandpass filtered at 10 Hz to 2000 Hz). EMG signals were continuously streamed by using LabChart software (LabChart 8.0) to monitor MEPs and epochs were recorded with a 150 ms window length covering from 50 ms before to 100 ms after TMS pulse. The “hot spot” corresponded to the scalp location where TMS intensity was sufficient to evoke a motor response (∼ 50 uV) in the FDI muscle, as compared to the APB muscle, in at least 50% of the trials.

### EEG

As reported in ^13^, whole scalp 64-channel EEG data was collected with a TMS-compatible amplifier system (actiCHamp system, Brain Products GmbH, Munich, Germany) and labeled in accordance with the extended 10–20 international system. EEG data were online referenced to Fp1 electrode. Electrode impedances were maintained below 5kΩ at a sampling rate of 1000 Hz. EEG signals were digitized using a BrainCHamp DC amplifier and linked to BrainVision Recorder software (version 1.21) for online monitoring. Digitized EEG electrode locations on the scalp are also co-registered to individual MRI scans using Brainsight™ TMS Frameless Navigation system.

### TMS targets

TMS targets were individualized using group-level resting-state functional networks maps extracting with a 7 networks parcellation ^32^ which divides the brain into visual (VISN), somatosensory (SMN), limbic (LIMN), dorsal attention (DAN), anterior salience (ASN), default mode (DMN), and fronto-parietal (FPN) RSNs. Confidence maps, created from over 1,000 young healthy adults, were used representing the estimate for each vertex across the cortical mantle as belonging to each of the 7 RSNs. To extrapolate individual TMS targets, group-level functional parcellations and confidence maps were used to constrain the search for the vertex with the most reliable peak within each network, with larger values indicating higher confidence. The 7-network functional cortical atlas and the confidence maps were firstly projected onto subject’s cortical surface using the spherical registration ^26^ and then were resampled to native structural T1w MRIs. The resampled individual confidence maps were used to select the weighted voxels with the highest confidence value in angular gyrus and superior parietal in the right hemisphere therefore picking DMN and DAN stimulation spots, respectively. For a visualization of the individual DAN and DMN see Supplementary Fig. S1.

### TMS-EEG data collection

At the beginning of each visit individual RMT was defined as the lowest stimulation intensity necessary to evoke a MEP (~ 50 uV) in at least 50% of the trials ^33^. The “hotspot” of stimulation was therefore determined as the cortical hand region were MEPs were larger and more consistent in the right FDI muscle, as compared to APB muscle ^34^. During the stimulation application, participants were asked to wear earplugs ^35^ where auditory white noise was played to minimize the impact of the TMS click ^36^. A thin layer of foam was placed under the TMS coil to minimize somatosensory contamination of the TMS-evoked EEG potentials. Each participant completed two identical experimental sessions 1 month apart were a total of 120 single TMS pulses were delivered to each stimulation target at an intensity of 120% RMT with randomly jittered (3000–5000 ms) inter stimulus intervals.

### EEG data processing

A customize script running in Matlab R2017b (Math-Works Inc., USA) was used for the offline data preprocessing mainly performed by EEGLAB 14.1 toolbox ^37^. A single block of 120 trials was firstly created by margining the two single blocks of 60 trials each, and then segmented into epochs of 1500 ms each (from − 500 ms (pre-pulse) to 1000 ms (post-pulse)). Baseline correction was performed using an amplitude of the mean pre-pulse (− 500 ms to − 100 ms) signal and raw data were visually inspected to then remove noisy channels. Zero-padding was applied on a window of − 2 ms to 14 ms to reject early TMS pulse artefact and noisy epochs were then removed based on the voltage (≥ 100 μV), kurtosis (≥ 3), joint probability (single channel-based threshold ≥ 3.5sd) and visual inspection. In order to minimize overfitting and noise components, the dimensionality of the data was firstly reduced to 60 components via principal component analyses (PCA). Following a first round of fast independent component analysis (fICA) ^38^ was run specifically aimed at removing remaining early TMS-evoked and EMG artefacts. A linear interpolation was used to interpolate the zero-padded time window and the EEG data were then band pass filtered using a forward–backward 4th order Butterworth filter from 1 to 100 Hz, notch filtered between 57 and 63 Hz, and referenced to global average. A second PCA was further employed to reduce the data dimensionality into 57 components before removing remaining artefact (e.g. eye movement/blink, muscle noise (EMG), single electrode noise, TMS-evoked muscle, cardiac beats, auditory evoked potentials) with a second round of fICA ^39^. During both fICA, the components were visually inspected where a semi-automated artefact detection algorithm incorporated into the open source TMS-EEG Signal Analyzer (TESA v0.1.0-beta; https://nigelrogasch.github.io/TESA/) was used ^39^. Finally, a low pass filtered with a 4th order Butterworth filter at 50 Hz was employed and previously removed channels were spherically interpolated. For an overview of the TMS-EEG data preprocessing and analysis please see Supplementary Fig. S4.

### EEG source reconstruction

All TMS-evoked EEG source reconstruction was performed in Brainstorm ^40^. First, FreeSurfer was used to extract the cortical envelope from a T1w which was then imported into Brainstorm using the individual anatomical landmarks extracted from Brainsight™ (nasion ‘NAS’, left pre-auricular ‘LPA’, and right pre-auricular ‘RPA’ points). Realistically shaped surface meshes of the brain, skull and scalp were extracted from the provided anatomical image using the default number of 1922 vertices per layer. The cortical surface distributed with Brainstorm was downsampled to around 1500 vertices.

Next, the EEG epochs, − 500 ms to 1000 ms with respect to TMS pulse, for each TMS trial were uploaded and average epoch time series was generated for each subject. Forward model solution of neuro electric fields was performed using the open MEEG symmetric boundary element method ^41^, all with default parameter settings ^40^. Noise covariance was estimated from individual trials using the pre TMS (− 500 ms to 0 ms) time window as baseline. The inverse model solution of the cortical sources was performed using the minimum norm estimation (MNE) method with current density ^42^ and constraining source dipoles to the cortical surface. The resulting output of EEG source reconstruction was the MNE current density time series for each cortical voxel.

### Source-level metrics

In order to determinate which between functional and structural connectivity mostly predict the TMS-induced signal propagation, the average current density timeseries were extracted from the DWI and fMRI gray matter projections. The final timeseries were normalized (z-scores), rectified ^17^ and the difference between the signal extracted from DWI and fMRI projection map was computed. In order to control for type I error, this result was compared with 1000 of surrogate differences obtained by performing a condition-wise permutation testing ^43^ with a significance threshold set at p < 0.05. Then, a cluster-based thresholding ^19^ was performed retaining only clusters that exceeded the 95th percentile of the distributions of contiguous significant time points obtained for all the permutations. Finally, the area under the curve (AUC) was extracted from the significant clusters with positive and negative values indicating that the TMS-EEG signal propagates more on DWI or fMRI projection map, respectively.

## Results

### Structural vs functional signal propagation

**Figure 2:**
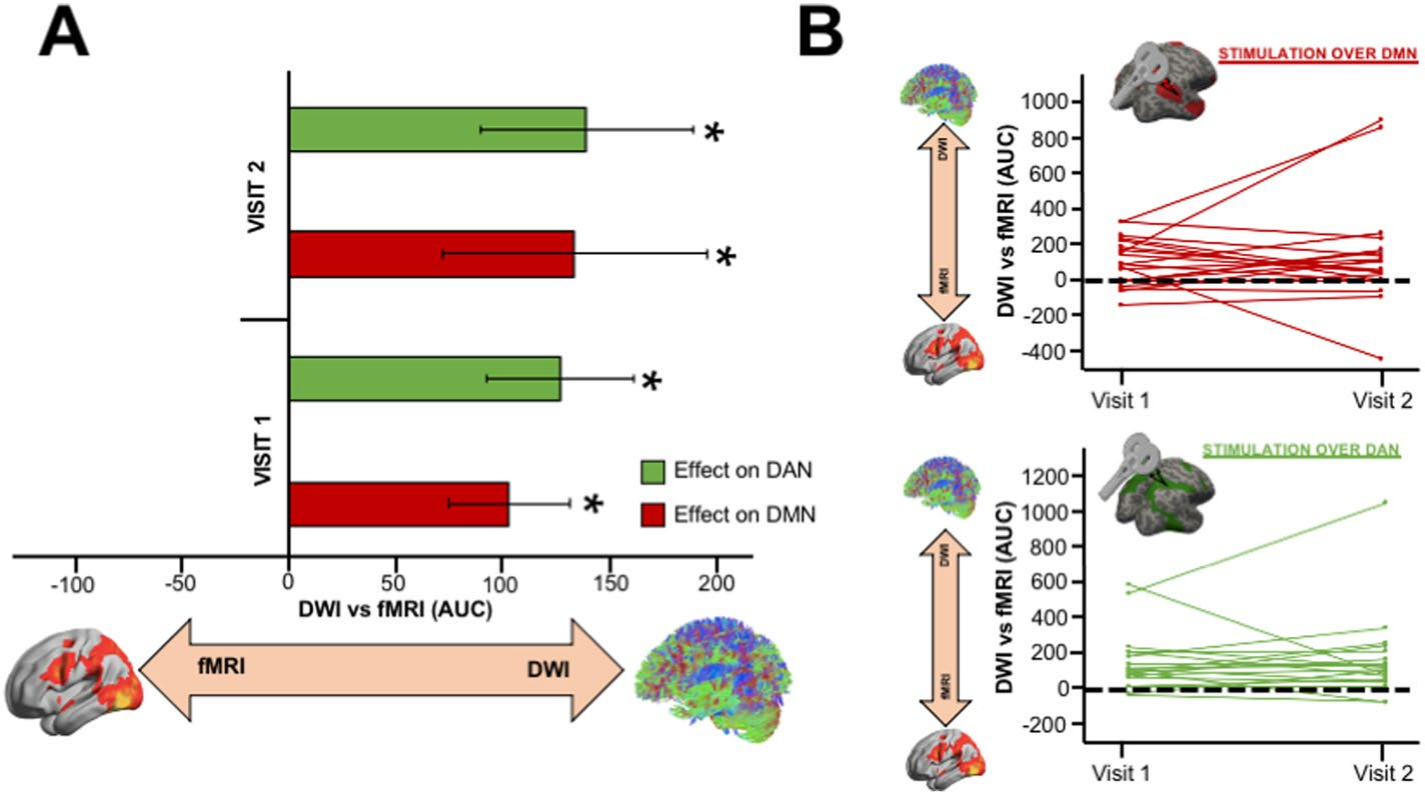
Propagation of the TMS-induced signal within DWI and fMRI projection maps. (**A**) Grand mean average and standard error of the mean (SEM) of the AUC extracted from DWI and fMRI projection maps. Significant positive values were found for both stimulation condition across visits indicating that the TMS-induced signal propagated more on DWI (rather than fMRI) projection map (Visit 1: DMN: average = 103.56, SEM = 28.26; Visit 2: DMN: average = 134.28, SEM = 62.02; Visit 1: DAN: average = 127.16, SEM = 34.48; Visit 2: DAN: average = 139.83, SEM = 49.86). (**B**) Test–retest reliability of the AUC extracted comparing the signal within DWI and fMRI projection maps. A high reproducibility across visits were found for both DMN (top, red lines) and DAN (bottom, green lines).

To test whether the propagation/spread of TMS-induced potentials is better addressed by structural or functional connected cortical regions, the source reconstructed signals extracted from DWI and fMRI projection map were statistically compared across subjects. After 1000 permutation tests, the AUC was finally computed from the significant clusters within the 350 ms post-TMS period, with positive and negative values indicating that the TMS-EEG signal propagated more on DWI or fMRI projection map, respectively. Significant positive values (Fig. 2A) were found for both stimulation condition across visits indicating that the TMS-induced signal propagation is better addressed by DWI (rather than fMRI) projection map (Visit 1: DMN: average = 103.56, SEM = 28.26; Visit 2: DMN: average = 134.28, SEM = 62.02; Visit 1: DAN: average = 127.16, SEM = 34.48; Visit 2: DAN: average = 139.83, SEM = 49.86).

Remarkably, the AUC extracted from significant clusters were highly reproducible (Fig. 2B) within each participant and across visits indicating the reliability of TMS-evoked cortical activation dynamics.

### Relationship between structural connectivity and resting-state EEG

**Figure 3:**
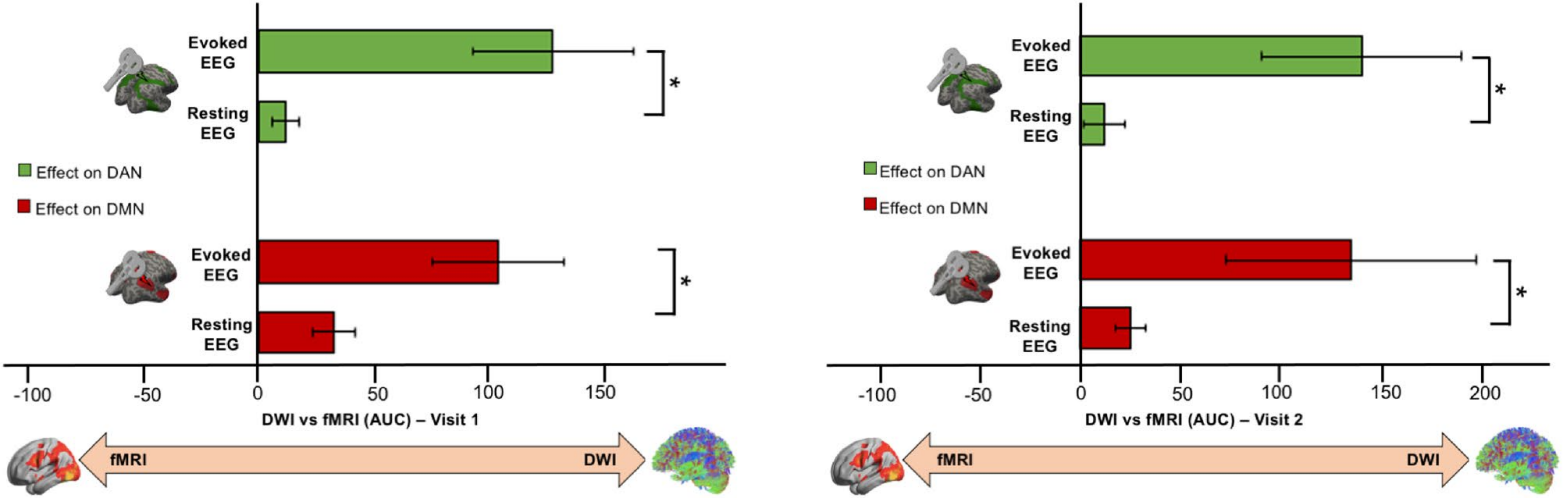
Spontaneous vs Evoked EEG activity. TMS-evoked signal propagation was significatively higher compare to resting-state EEG activity in structurally connected regions for both DMN (red bar) and DAN (green bar).

In order to demonstrate that the aforementioned results were not a function of resting-state brain oscillations, but due to specific activity elicited by TMS instead, source-level activity was computed (i.e. AUC) for both DWI and fMRI projection maps using eyes-open resting-state EEG data. For both stimulation conditions, the TMS-induced potential propagation was better addressed by structural—rather than functional—connected cortical regions. Such pattern was found for both TMS-evoked and resting-state EEG activity, even though was significatively higher for the former (Fig. 3).

### Structural connectivity profile

**Figure 4:**
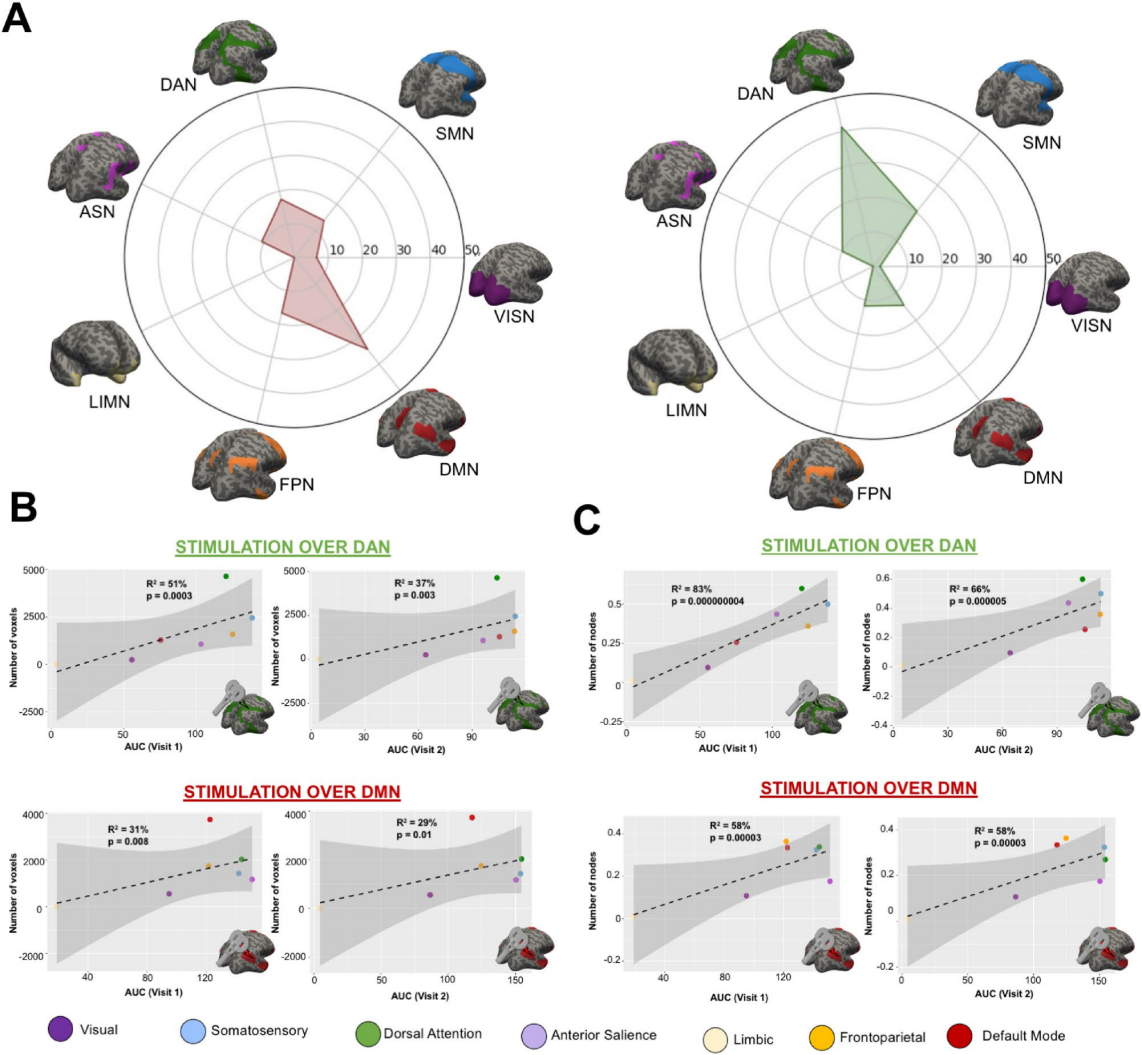
Relationship between source TMS-EEG signal and DWI pathway projection. (**A**) Percentage of grey matter voxels (divided by network) reached by seed-based tractography from the stimulation spot. As for angular gyrus (DMN stimulation seed), the white matter fibers not only reach cortical regions belonging to the DMN (34.52%) but also affect the other RSNs (VISN = 6.52%; SMN = 13.95%; DAN = 17.55%; ASN = 10.72%; LIMN = 0.04%; FPN = 16.71%). Likewise for the superior parietal lobule (DAN stimulation spot), the seed-based tractography do not involve grey matter voxels belonging to DAN (41.38%), but also reach the other RSNs (VISN = 2.00%; SMN = 20.42%; ASN = 9.98%; LIMN = 0.01%; FPN = 11.77%; DMN = 14.45%). (**B**) A significant positive correlation was found between the number of voxels reached by the seed-based tractography (divided by network) and the AUC extracted for both DAN (visit 1: R^2^ = 51%, p = 0.0003; visit 2: R^2^ = 37%, p = 0.003) and DMN (visit 1: R^2^ = 31%, p = 0.008; visit 2: R^2^ = 29%, p = 0.01). (**C**) A significant positive correlation was found between the number of networks nodes (normalized for the overall number of network nodes) reached by the seed-based tractography and the AUC extracted for both DAN (visit 1: R^2^ = 82%, p = 0.000000004; visit 2: R^2^ = 66%, p = 0.000005) and DMN (visit 1: R^2^ = 58%, p = 0.00003; visit 2: R^2^ = 58%, p = 0.00003). Scatterplots were generated using R software (https://www.R-project.org/) ^44^ version 1.1.453.

Given the aforementioned result demonstrating how TMS-EEG signal reach mainly structural connected regions, the interaction between the individual structural connectome and the response to TMS were further investigated. Firstly, the individual DWI projection maps were divided following the 7 networks parcellation in order to disentangle whether if the white matter fibers would reach mainly cortical regions belonging to the network engaged. As for angular gyrus (DMN stimulation seed, Fig. 4A left), the white matter fibers were not constrained to cortical regions belonging to the DMN (34.52%) but also extended to the other RSNs (VISN = 6.52%; SMN = 13.95%; DAN = 17.55%; ASN = 10.72%; LIMN = 0.04%; FPN = 16.71%). Likewise, for the superior parietal lobule (DAN stimulation spot, Fig. 4A right), the seed-based tractography not only reach grey matter voxels belonging to DAN (41.38%), but also affect the other RSNs (VISN = 2.00%; SMN = 20.42%; ASN = 9.98%; LIMN = 0.01%; FPN = 11.77%; DMN = 14.45%).

Then, in order to investigated whether if the size and the number of regions reached by the seed-based tractography would linearly determinate the TMS-EEG response, the AUC was calculated for each network clusters of the DWI projection maps. A significant positive correlation was found (Fig. 4B) between the number of voxels reached by the seed-based tractography (divided by network) and the AUC extracted for both DAN (visit 1: R2 = 51%, p = 0.0003; visit 2: R^2^ = 37%, p = 0.003) and DMN (visit 1: R^2^ = 31%, p = 0.008; visit 2: R^2^ = 29%, p = 0.01). Moreover, a significant positive correlation was found (Fig. 4C) between the number of networks nodes (normalized for the overall number of nodes per network) reached by the seed-based tractography and the AUC extracted for both DAN (visit 1: R^2^ = 82%, p = 0.000000004; visit 2: R^2^ = 66%, p = 0.000005) and DMN (visit 1: R^2^ = 58%, p = 0.00003; visit 2: R^2^ = 58%, p = 0.00003).

## Discussion

A recent study by our group has used a network-perturbation approach to characterize individual brain dynamics within discrete brain networks with high temporal resolution ^13^, demonstrating how intrinsic network structural connectivity provides valuable information to estimate network engagement. Here, we further expand this concept by directly comparing the signal extracted from brain regions either functionally or structurally connected to the stimulation site. Our findings support that white matter pathways are better in addressing the propagation of the action potentials induced by an external perturbation, compared to functionally connected brain regions. Such result was replicable across two separate visits and for two networks, supporting the relevance of macroscale structural connectivity in predicting network-level response to TMS perturbation. Finally, brain structural wiring seems helpful in predicting propagation of activity after a perturbation, but not during spontaneous resting-state activity, suggesting perturbation-based approaches as a valuable tool to investigate the the human connectome.

### TMS-induced propagation is better explained by structural metrics

In line with our expectations, the action potentials induced by an external perturbation mainly propagates within structurally rather than functionally connected regions, nominating the white matter pathways as the principal conductor for TMS-induced signal propagation.

Several experimental and computational studies in modern neuroscience aimed at investigating how rs-FC emerges from the brain's anatomical connections for a better understanding of the structure/function relationship ^45^. Indeed, it has been demonstrated how rs-FC arises and reflect underlying structural anatomy ^46^ even though the inverse relation is not straightforward, given that a functional dependency has also been observed between regions where there is little or no structural connectivity, possibly because of third regions that may act as functional links ^47^.

In the context of Noninvasive Brain Stimulation (NIBS), previous studies in animals and humans have shown how TMS influences local as well as distant cortical regions through their structural connectivity pathways ^3,48^, such as that the nature of the effects has been found strongly affected by the richness of the anatomical connectivity between regions and the number of synaptic steps ^6^. Using rs-FC measures, Eldaief and colleagues have demonstrated how the stimulation of a DMN node (i.e. the posterior cingulate cortex) allowed to indirectly modulate the whole network with divergent effects depending on stimulation conditions ^11^. Moreover, rs-FC has been employed to guide therapeutic TMS approaches in clinical settings, with few studies reporting promising results ^49^ and others showing no clinical improvements ^50^. Further studies have demonstrated how the induced TMS signal propagates within functionally connected local and distal regions belonging to the same network ^12^, with concurrent TMS-EEG-fMRI data showing how strong pre-TMS alpha spectral power reduces TMS-evoked hemodynamic activations within the motor system ^15^.

On the other hand, multiple studies have addressed the importance of white matter microstructural integrity for the propagation of externally induced action potentials ^51^ with further evaluation of the relationship between functional and structural connectivity using source estimated EEG recording and probabilistic tractography ^52^. A recent study has used structural connectivity to identify subject-specific targets in the middle frontal gyrus that were connected to a particular portion of the primary somatosensory cortex, demonstrating how TMS applied to this region improved tactile working memory whilst TMS to non-connected portions of the middle frontal gyrus—located just 18 mm away—did not ^53^. Moreover, structural connectivity has been shown to be a reliable and accurate technique for mapping of the course of subcortical pyramidal tracts during intraoperative MEPs evaluation, preserving motor function in patients with gliomas near the pyramidal tracts ^54^.

In line with previous animal study ^55^, our results demonstrated that white matter pathways better capture the spreading of the action potentials generated by sites structurally connected to the stimulation spot. Such results offer insight for the selection of optimal stimulation targets for maximal network engagement, as well as for the prediction TMS response in clinical populations. It is important to note that connectivity assessed with either technique (rs-fMRI, DWI) involves polysynaptic connections. Indeed, the resolution of DTI is generally not high enough to achieve monosynaptic fiber tracking in vivo ^56^, and rs-FC has been reported between regions in the monkey visual system with no direct anatomical connections ^57^, implying polysynaptic transmission. Related to this, a recent paper published by our group has demonstrated how TMS-induced signal mainly propagates within the stimulated network ^12^, even though changes in functional connectivity have also been found in other non-stimulated resting state networks reached via polysynaptic connection ^58^. In this framework, the present study might encourage the combination of DWI and fMRI data for target selection in order to optimize the engagement within the network of interest. However, prospective studies are needed to causally compare the effects of an external perturbation in a brain region identified using a standard vs a DWI-constrained functional connectivity approach.

### Perturbing the brain unveils underlying structural/functional dynamics

The aforementioned pattern, where action potentials spreading induced by an external perturbation was better detected by structurally (rather than functionally) connected regions, was not evident when analyzing resting-state EEG data. So far, multiple studies have addressed the importance of unconstrained spontaneous activity data for predicting individual cognitive functions ^59^, personality traits ^60^ and behavior ^61^. Conversely, recent evidences have shown how models built from task-based neuroimaging outperform models built from resting-state data alone in predicting individual cognitive abilities and connectivity patterns ^62,63^. Interestingly, a recent study published by our group ^12^ reported a significant positive correlation between individual fluid intelligence and the network engagement following an external perturbation, which was not observed when considering resting-state EEG. In line with this evidence, the present study supports the notion that TMS might be employed to unveil the underlying propagation dynamics of action potentials, suggesting perturbation-based approaches as a promising tool to investigate structure/function relationship in the human brain.

### Specificity of structural connectome

Given that the signal induced by an external perturbation propagates more on white matter pathways (rather than functionally connected regions), the interaction between the individual structural connectome and the response to TMS were further investigated. Even though the fiber bundles originated from the stimulated spots mostly reached cortical brain regions belonging to the same network, a conspicuous engagement of the other RSNs was also evident. Specifically, white matter tracts originating from the DMN stimulation seed also reached regions belonging to FPN, whilst fiber pathways originating from the DAN also engaged the SMN network. Interestingly, the negative correlation ^10^ and causal interaction ^64^ between the FPN and the DMN has already been shown in the context of TMS. Indeed, aberrant functional connectivity patterns in the dynamics of these two networks have been associated with individual variability in multiple cognitive functions, motor behaviors, and symptomatology of various neurological and psychiatric disorders ^65^. Furthermore, reduced functional connectivity has been demonstrated between the DAN and the SMN in late onset depression compared to healthy controls ^66^. Remarkably, the AUC calculated for each network cluster of the DWI projection maps was predicted by both the size and the number of nodes reached by the seed-based tractography, showing once again the importance of structural connectivity for the linear determination of the system response.

### Limitations of the study and future directions

Our results must be interpreted within the debate about the spatial resolution of source localization methods. Indeed, even though we observed distinct propagation patterns depending on the network being stimulated, a more precise spatial mapping of TMS-induced network effects would be better captured by combining TMS-EEG with concurrent fMRI acquisitions ^15^. Moreover, it is well known that the neural impact of a TMS pulse is not only determined by the properties of that stimulus but also by the initial activation state of the perturbed region ^7^. Such state-dependency effect should not presumably affect the source-reconstructed EEG signal measured in brain areas structurally connected with the stimulation spot. On the other hand, in the context of fMRI, previous TMS/task-evoked studies have demonstrated how the effects of an external perturbation are highly dependent on the initial state of the stimulated region ^67^. Future studies should investigate the impact of ongoing brain oscillations on the effects of a TMS pulse.

As for diffusion signal, current tractography techniques are known to underestimate inter-hemispheric connectivity—which is substantial between homotopic brain regions—a limitation that has been mitigated by using probabilistic tractography with multiple fiber orientations ^68^. Furthermore, the b-value utilized for DWI in this study was relatively low, and diffusion data with higher angular resolution would improve accurate tract identification, especially in regions of crossing fibers ^69^. Moreover, given the importance of white matter pathways for the conduction of externally induced action potentials, future studies might employ quantitative MRI techniques combined with advanced biophysical models to measure microstructural features of white matter such as axon diameter, the g-ratio and the overall tract length ^70^.

Furthermore, since pre-surgical neuronavigated TMS mapping has been successfully used to provide seed points for fiber tracking based on DWI data when reconstructing the corticospinal tract ^71^ as well as language-relevant pathways ^72^, future scenarios may even extend to the combination of DWI with dual-coil TMS approaches, combining measures of structural and effective connectivity.

## Conclusion

Even though stimulation of brain networks via TMS has been reported to induce activation in functionally connected regions, direct comparison with structural connectivity of the same target regions show a stronger propagation over cortical regions connected via white matter pathways. Additionally, results suggest DWI-based estimation of propagation patterns can be used to estimate off-target engagement of other resting-state networks and possibly guide target selection to maximize specificity in future studies.

## Supplementary Information

Supplementary Information.
