# Supplementary material for: Perturbation of resting-state network nodes preferentially propagates to structurally rather than functionally connected regions
Source: Sci Rep. 2021 Jun 14;11:12458. doi: 10.1038/s41598-021-90663-z (PMC8203778; doi:10.1038/s41598-021-90663-z)
Supplement: Supplementary file 1 — Supplementary Information. [file 41598_2021_90663_MOESM1_ESM.pdf]

Perturbation of resting-state network nodes preferentially propagates to structural rather than functional connected regions

Davide Momi[1,2], Recep A. Ozdemir[1], Ehsan Tadayon[1], Pierre Boucher[1], Alberto Di Domenico[3], Mirco Fasolo[3], Mouhsin M. Shafi[1], Alvaro Pascual-Leone[4,5,6], Emiliano Santarnecchi[1,7]

[1] Berenson-Allen Center for Non-Invasive Brain Stimulation, Beth Israel Deaconess Medical Center, Harvard Medical School, Boston, MA, USA
[2] Department of Neuroscience, Imaging and Clinical Sciences, University of Chieti-Pescara, Chieti
[3] Department of Psychological Science, Humanities and Territory, University of Chieti-Pescara, Chieti, Italy
[4] Hinda and Arthur Marcus Institute for Aging Research and Center for Memory Health, Hebrew Senior Life
[5] Department of Neurology, Harvard Medical School, Boston, MA, USA
[6] Guttmann Brain Health Institut, Guttmann Institut, Universitat Autonoma, Barcelona, Spain
[7] Siena Brain Investigation & Neuromodulation Lab (Si-BIN Lab), Department of Medicine, Surgery and Neuroscience, Neurology and Clinical Neurophysiology Section, University of Siena, Italy

Corresponding author:
Emiliano Santarnecchi
Berenson-Allen Center for Non-Invasive Brain Stimulation, Beth Israel Medical Center
Harvard Medical School, Boston, MA, USA
office +1-617-667-0326
mobile +1-617-516-9516
esantarn@bidmc.harvard.edu

1. SUPPLEMENTARY MATERIAL AND METHODS

1.1 Generating individual motor network maps

1.2 Modelling of the TMS-induced electric field

1.3 Diffusion Tensor Imaging (DTI) metrics extraction

2. SUPPLEMENTARY RESULTS

2.1 DAN and DMN white matter bundles properties

2.2 Relationship between DTI metrics and the AUC

1. SUPPLEMENTARY MATERIAL AND METHODS

1.1 Generating individual motor network maps

First, group-average network functional cortical atlas [1] and confidence maps were morphed into the subject's cortical surface using surface-based registration [2] Following, the Individualized DAN and DMN network were extracted and projected to native structural T1w MRIs (Figure S1). To extrapolate individual TMS targets, the resampled individual confidence maps were used to select the weighted voxels with the highest confidence value in angular gyrus and superior parietal in the right hemisphere therefore picking DMN and DAN stimulation spots, respectively.

1.2 Modelling of the TMS-induced electric field

A tetrahedral head model (mesh file) was created using SimNIBS [3], consisting of five tissue types: white matter (WM), grey matter (GM), cerebro-spinal fluid (CSF), skull, and scalp. The assigned conductivity values were fixed: 0.126 S/m (WM), 0.275 S/m (GM), 1.654 S/m (CSF), 0.01 S/m (skull), 0.465 S/m (scalp). The distance between the coil and the cortex was set to 10 mm, as measured in our MRI images, and the coil handle was oriented following the coordinates used in the actual experiment using pynetstim (https://github.com/EhsanTadayon/pynetstim). The rate of change of the coil current (dI/dt) was calculated quasi-static regime [4], according to the following equation:

$$E = -\frac{\vartheta A}{\vartheta t} - \Delta\varphi$$

where E is the electric field vector and φ denotes the electric potential. Given that in literature there is no consensus on how selectively identify only the neural tissue recruited by the TMS pulse [5], we defined the point with maximal E-field and from there created a sphere of radius 0.5cm.

1.3 Diffusion Tensor Imaging (DTI) metrics extraction

Commonly used DTI maps were generated using FDT [6] including mean diffusivity (MD), fractional anisotropy (FA), axial diffusivity (AD) and radial diffusivity (RD). These measures are directly related to the value of the three main eigenvalues of the tensor (λ1, λ2, λ3) and represent the value of the displacement/diffusion for each specific vector.

FA indicates the overall directionality of water diffusion that is greater in organized white matter tracts and lower in CSF and disorganized fiber.

$$FA = \sqrt{\frac{(\lambda 1-\lambda 2)^2 + (\lambda 2-\lambda 3)^2 + (\lambda 1-\lambda 3)^2}{2(\lambda 1^2 + \lambda 2^2 + \lambda 3^2)}}$$

While FA is highly sensitive to microstructural changes, it is not very specific to the nature of change. On the other hand, MD describes the rotationally invariant magnitude of water diffusion within brain tissue.

$$MD = \frac{(\lambda 1 + \lambda 2 + \lambda 3)}{3}$$

It is an inverse measure of the membrane density, being very similar for both GM and WM and higher for CSF and sensitive to cellularity, edema, and necrosis.

RD represents the apparent water diffusion coefficient in the direction perpendicular to the axonal fibers.

$$RD = \frac{(\lambda 2 + \lambda 3)}{2}$$

RD could be considered a parameter of demyelination or glia cell impairment being also affected by changes in the axonal diameters or density [7].

Finally, AD is a measure of water diffusion along the principal axis of diffusion and represents a biomarker of the axonal integrity and conductivity.

$$DA = \lambda 1$$

Taken together, all these measures reflects the degree to which diffusion of water molecules is restricted by microstructural elements such as cell bodies, axons, myelin, and other constituents of cytoskeleton [8].

This metrics were extracted for the DAN and DMN structural connectivity profile correlated with the AUC extracted from DWI and fMRI projection maps differences.

2. SUPPLEMENTARY RESULTS

2.1 DAN and DMN white matter bundles properties

Seed-based anatomically constrained tractography [9] was performed in order to extrapolate the white matter bundle using the thresholded E-field DMN and DAN ROIs as seed. On average the DAN tract (Figure S2) was 205.04±26.349mm and the following DTI metrics stats: AD= 1.23±0.05; FA=0.46±0.05; MD=0.79±0.03; RD=0.58±0.04. As for the DMN tract (Figure S3), the average length was 221.04±32.346mm and the following DTI metrics stats: AD= 1.20±0.06; FA=0.45±0.03; MD=0.78±0.03; RD=0.58±0.03.

2.2 Relationship between DTI metrics and the AUC

As shown in Figure S5, DTI metrics (AD, FA, MD, RD) were not significantly related with the AUC for both DAN (visit 1: AD: R2=0.02%, p=0.46; FA: R2=0.02%, p=0.45; MD: R2=0.01%, p=0.56; RD: R2=0.004%, p=0.76; visit 2: AD: R2=0.006%, p=0.71; FA: R2=0.001%, p=0.87; MD: R2=0.02%, p=0.49; RD: R2=0.008%, p=0.68) and DMN (visit 1: AD: R2=0.05%, p=0.27; FA: R2=0.04%, p=0.32; MD: R2=0.004%, p=0.75; RD: R2=0.08%, p=0.69; visit 2: AD: R2=0.01%, p=0.57; FA: R2=0.08%, p=0.69; MD: R2=0.01%, p=0.62; RD: R2=0.00001%, p=0.99).

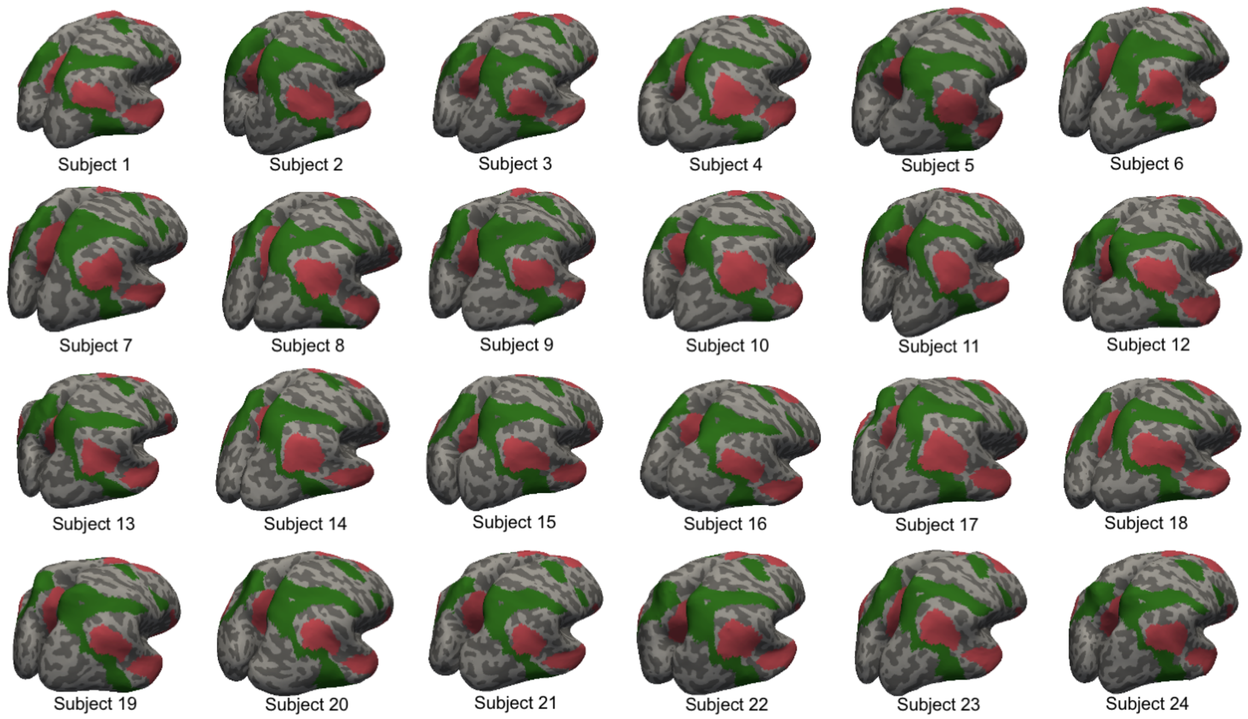

Figure S1. Individuals' DAN and DMN Networks functional cortical atlas. Individualized DAN (green) and DMN (red) extracted and projected to native structural T1w MRIs. FreeSurfer (https://surfer.nmr.mgh.harvard.edu/) [2] version 6.0 was used to generate this figure.

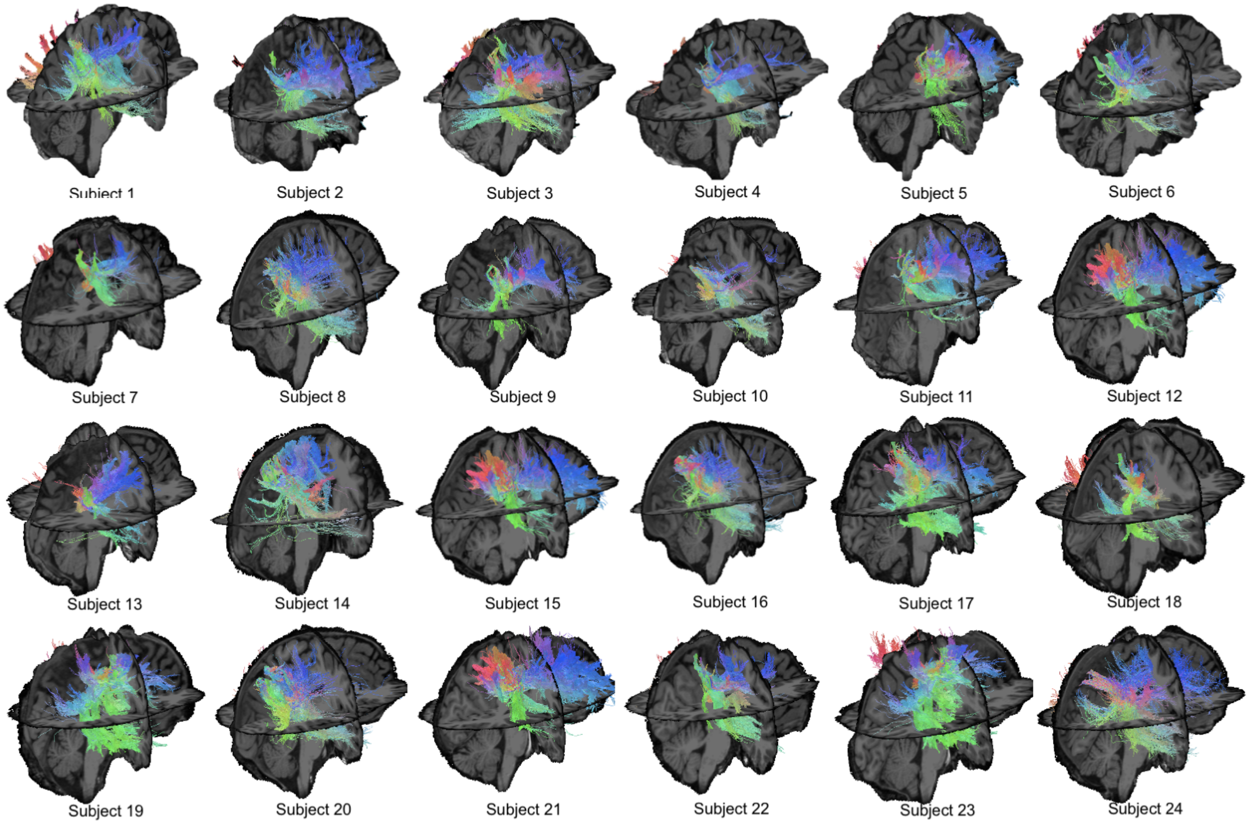

Figure S2. Individuals' white matter bundle using the DAN stimulation spot as seed. Seed-based anatomically constrained tractography [9] was performed in order to extrapolate the white matter bundle using the DAN E-field map as seed. MRtrix3 (www.MRtrix.readthedocs.io) [10] version 3.0 was used to generate this figure.

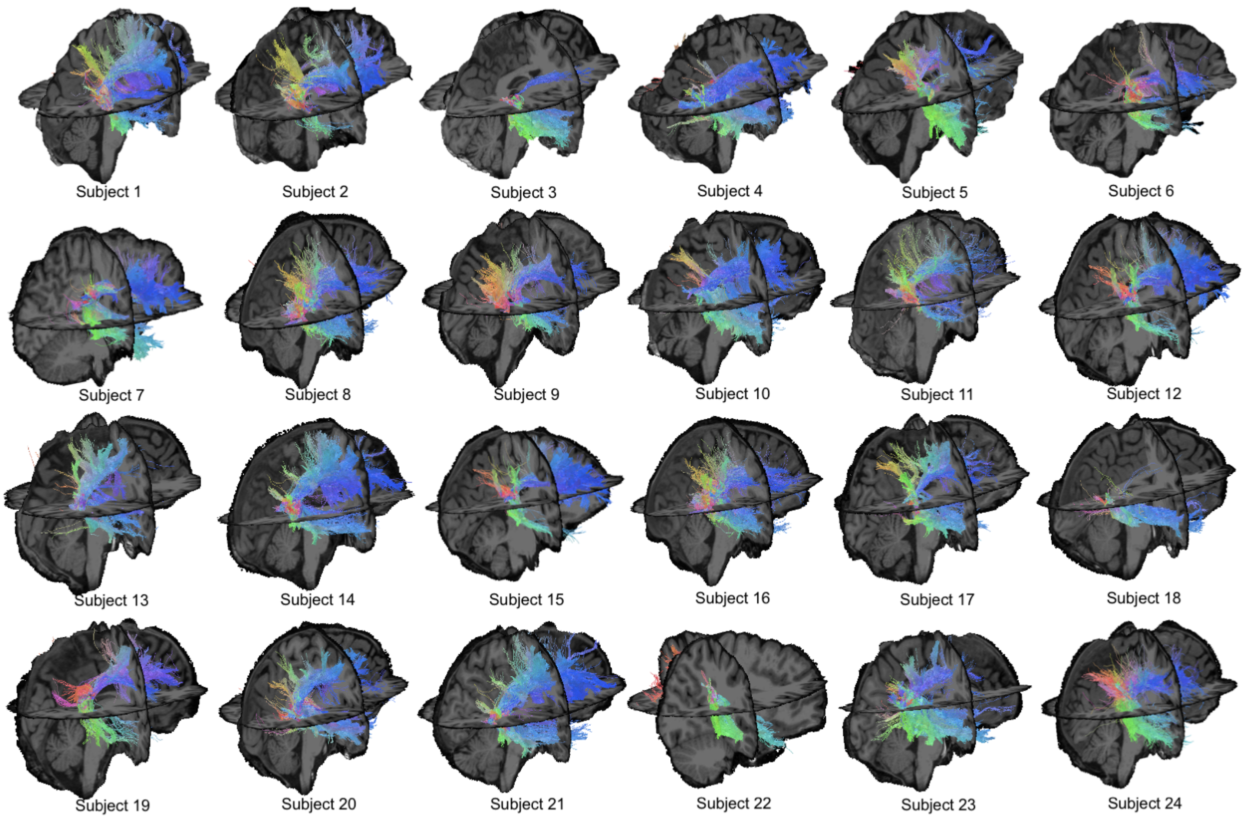

Figure S3. Individuals' white matter bundle using the DMN stimulation spot as seed. Seed-based anatomically constrained tractography [9] was performed in order to extrapolate the white matter bundle using the DMN E-field map as seed. MRtrix3 (www.MRtrix.readthedocs.io) [10] version 3.0 was used to generate this figure.

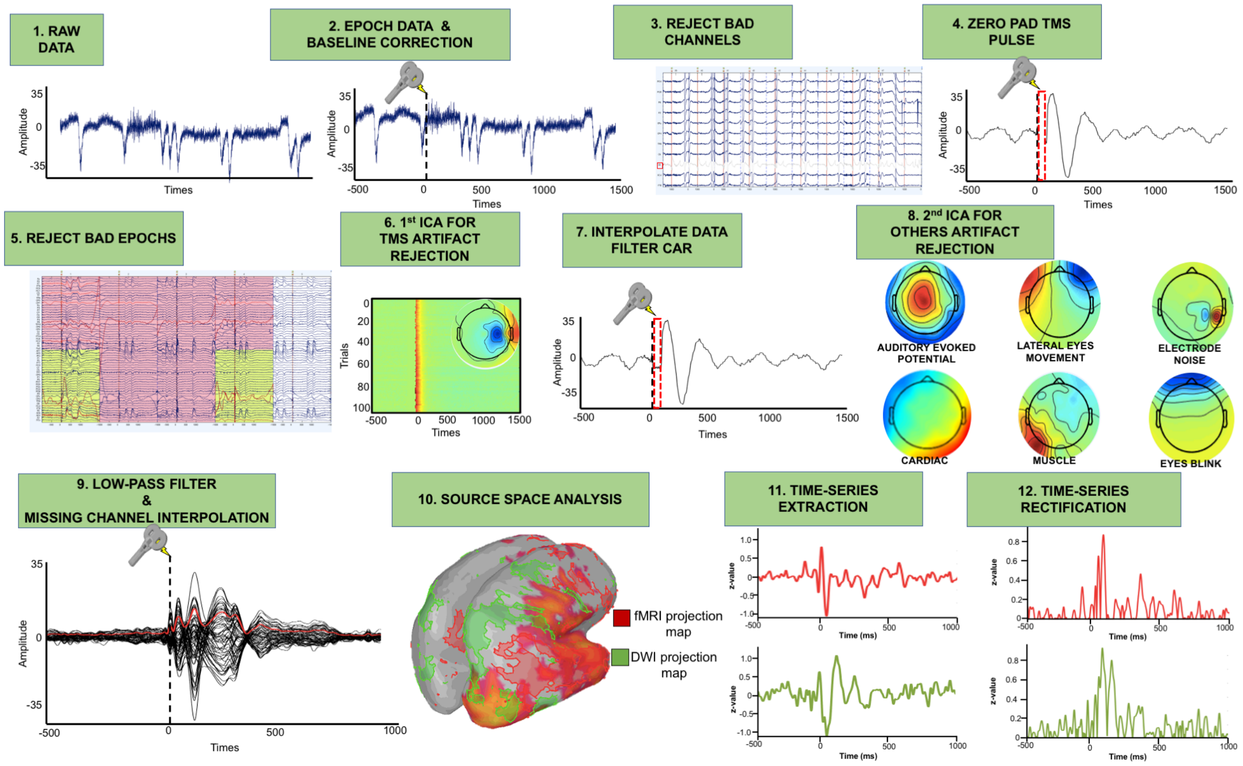

Figure S4. EEG Signal Preprocessing and Analysis. Major steps of EEG data cleaning procedures on a sample data set and resulting signal transformation showing evolution of TMS evoked potentials (TEPs), with final TMS evoked potential (step 9). The preprocessed signal was then used for source space analysis (step 10) and finally time-series from the DWI and fMRI projection maps were extracted (step 11) and rectified (step 12). See methods for details. No software was used to generate this figure.

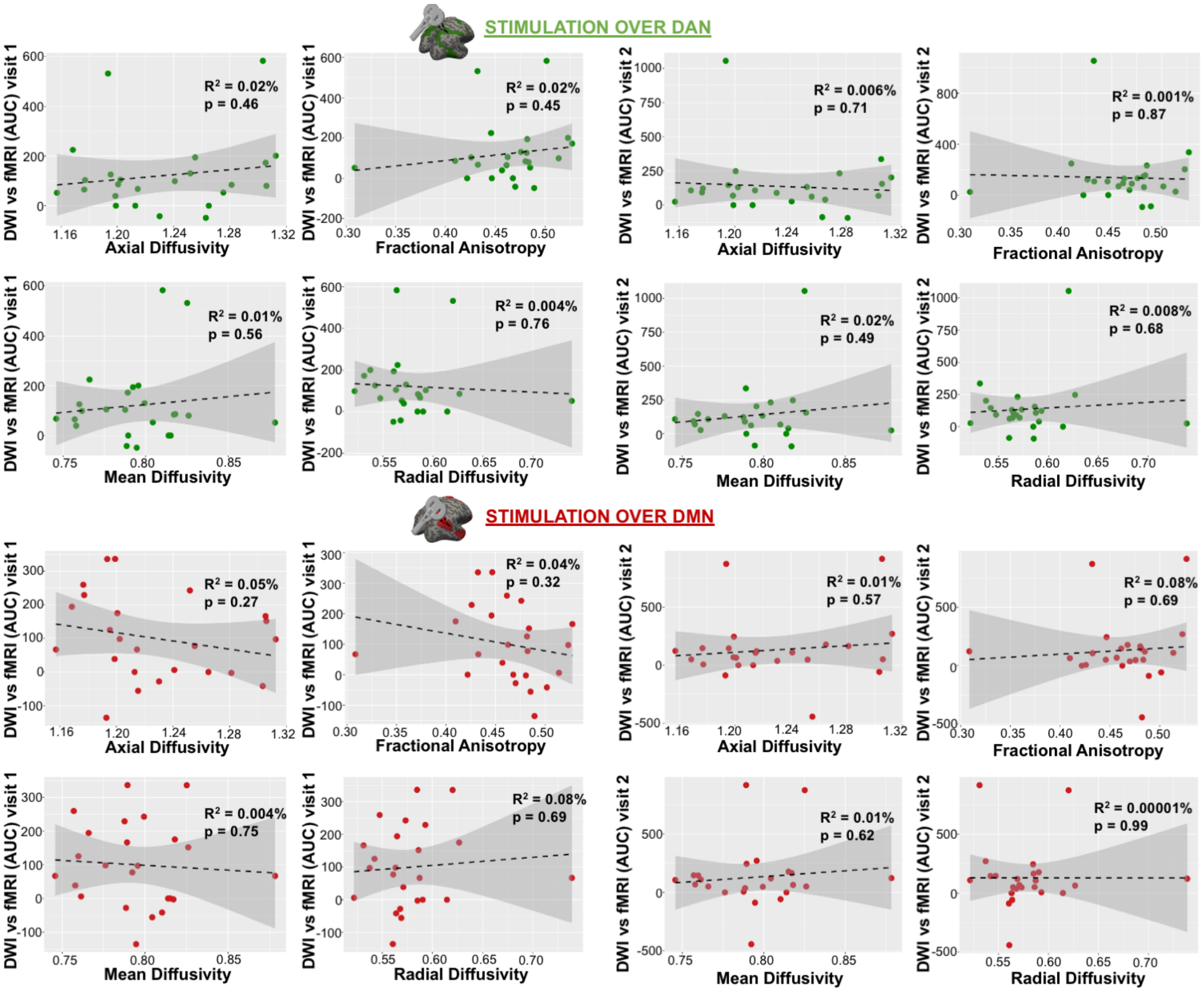

Figure S5. Relationship between DTI metrics and the AUC for DAN and DMN. DTI metrics (AD, FA, MD, RD) were not significantly related with the AUC for both DAN (visit 1: AD: R2=0.02%, p=0.46; FA: R2=0.02%, p=0.45; MD: R2=0.01%, p=0.56; RD: R2=0.004%, p=0.76; visit 2: AD: R2=0.006%, p=0.71; FA: R2=0.001%, p=0.87; MD: R2=0.02%, p=0.49; RD: R2=0.008%, p=0.68) and DMN (visit 1: AD: R2=0.05%, p=0.27; FA: R2=0.04%, p=0.32; MD: R2=0.004%, p=0.75; RD: R2=0.08%, p=0.69; visit 2: AD: R2=0.01%, p=0.57; FA: R2=0.08%, p=0.69; MD: R2=0.01%, p=0.62; RD: R2=0.00001%, p=0.99).

Scatterplots were generated using R software (https://www.R-project.org/) [11] version 1.1.453.

Supplementary References

1. Yeo, B. T. T. *et al.* The organization of the human cerebral cortex estimated by intrinsic functional connectivity. *J. Neurophysiol.* **106**, 1125–1165 (2011).

2.Fischl, B. *et al.* Automatically parcellating the human cerebral cortex. *Cereb. Cortex N. Y. N 1991* **14**, 11–22 (2004).

3. Thielscher, A., Antunes, A. & Saturnino, G. B. Field modeling for transcranial magnetic stimulation: A useful tool to understand the physiological effects of TMS? in *2015 37th Annual International Conference of the IEEE Engineering in Medicine and Biology Society (EMBC)* 222–225 (IEEE, 2015). doi:10.1109/EMBC.2015.7318340.

4. Opitz, A., Paulus, W., Will, S., Antunes, A. & Thielscher, A. Determinants of the electric field during transcranial direct current stimulation. *NeuroImage* **109**, 140–150 (2015).

5. Romero, M. C., Davare, M., Armendariz, M. & Janssen, P. Neural effects of transcranial magnetic stimulation at the single-cell level. *Nat. Commun.* **10**, 2642 (2019).

6. Behrens, T. E. J. *et al.* Characterization and propagation of uncertainty in diffusion-weighted MR imaging. *Magn. Reson. Med.* **50**, 1077–1088 (2003).

7. Basser, P. J., Mattiello, J. & LeBihan, D. MR diffusion tensor spectroscopy and imaging. *Biophys. J.* **66**, 259–267 (1994).

8. Beaulieu, C. The basis of anisotropic water diffusion in the nervous system - a technical review. *NMR Biomed.* **15**, 435–455 (2002).

9. Smith, R. E., Tournier, J.-D., Calamante, F. & Connelly, A. Anatomically-constrained tractography: improved diffusion MRI streamlines tractography through effective use of anatomical information. *NeuroImage* **62**, 1924–1938 (2012).

10. Tournier, J.-D., Calamante, F. & Connelly, A. MRtrix: Diffusion tractography in crossing fiber regions. *Int. J. Imaging Syst. Technol.* **22**, 53–66 (2012).
11. R Core Team. *R: A Language and Environment for Statistical Computing*. (2018).
